# Association of revised morphological uterus sonographic assessment (MUSA) features of adenomyosis and IVF outcomes

**DOI:** 10.1038/s41598-026-50999-w

**Published:** 2026-04-30

**Authors:** Soo Jin Han, Je Yeon Lee, Hye-Ok Kim, You Shin Kim, Tae Ki Yoon

**Affiliations:** 1 Department of Obstetrics and Gynecology, CHA University Fertility Center Seoul Station, Seoul, South Korea; 2 Department of Obstetrics and Gynecology, Seoul National University College of Medicine, Seoul, South Korea; 3 Department of Obstetrics and Gynecology, Ewha Womans University College of Medicine, Seoul, 07985 South Korea

**Keywords:** Adenomyosis, Assisted reproduction, Infertility, Ultrasound, Diseases, Endocrinology, Health care, Medical research

## Abstract

This study aimed to investigate the association between sonographic features of adenomyosis defined by the revised MUSA criteria and pregnancy outcomes in women undergoing in vitro fertilization (IVF) and embryo transfer (ET). All autologous ET cycles at a university-affiliated infertility center in Seoul, South Korea, between May 2022 and January 2024 were included in this study. All patients undergoing IVF were examined by the ultrasound strictly adhering to the revised MUSA definition at the time of examination. Patients with sonographic features of adenomyosis were assigned to the adenomyosis group (*n* = 300). During the same period, an age-matched 1:1 control group (*n* = 300) was selected from women who underwent embryo transfer and had no sonographic features of adenomyosis. Logistic regression analysis and generalized estimating equations were used to evaluate which specific sonographic features of adenomyosis are associated with pregnancy outcomes. Among the MUSA features, the interrupted junctional zone was the only independent factor significantly associated with a lower chance of clinical pregnancy (aRR = 0.75, 95% CI 0.58–0.95, *p* = 0.020), ongoing pregnancy (aRR = 0.72, 95% CI 0.52–0.99, *p* = 0.049), and an increased risk of biochemical pregnancy loss (aRR = 1.98, 95% CI 1.02–3.84, *p* = 0.044). This finding suggests that sonographic evaluation of the junctional zone should be considered in women with adenomyosis who desire pregnancy.

**Supplementary Information:**

The online version contains supplementary material available at 10.1038/s41598-026-50999-w.

## Introduction

Adenomyosis is a common gynecological disorder that can cause dysmenorrhea and menorrhagia, and its association with infertility remains a significant concern for reproductive-aged women. The reported prevalence of adenomyosis varies widely (5–70%) depending on the population studied because the diagnosis is primarily based on pathological findings post-hysterectomy^1,2^. Adenomyosis may impair embryo implantation by causing structural and functional defects in the endometrium and myometrial junctional zone (JZ)^3^. Previous studies reported that adenomyosis was observed in about 10% of subfertile women^4^, and the success rate of in vitro fertilization (IVF) decreases by 28% in cases with adenomyosis^5^. Several studies have reported a higher risk of miscarriage and impaired implantation in association with adenomyosis; however, the association remains controversial^4,6^.

From a pathophysiological perspective, adenomyosis is characterized by the presence of endometrial glands and stroma within the uterine myometrium. However, a pathophysiological diagnosis is practically not feasible for infertility patients who are not considered for hysterectomy. Consequently, the importance of diagnostic approaches has increased, and transvaginal sonography (TVS) is regarded as the first-line diagnostic method^7^. A meta-analysis reported TVS sensitivity of 74–78% and specificity of 76–88%, but variability in diagnostic criteria among the studies remains a limitation^8^. To address this, the Morphological Uterus Sonographic Assessment (MUSA) group proposed standardized ultrasonographic diagnostic criteria in 2015^9^, which were revised in 2022^7^. These guidelines emphasize that adenomyosis should only be diagnosed when direct features aligning with its pathophysiological findings are present.

For standardized diagnostic criteria to have clinical significance, their correlation with symptoms and prognosis must be established. However, the association between the revised MUSA criteria and fertility outcomes remains unclear. Adenomyosis is most commonly diagnosed in women in their forties. With the trend of women postponing their first pregnancy until their late thirties or early forties, the significance of adenomyosis regarding subfertility has increased.

In this study, we observed the association between each individual ultrasonographic feature of adenomyosis and pregnancy outcomes in women undergoing in vitro fertilization and embryo transfer. We aimed to determine which specific sonographic features of adenomyosis are particularly associated with fertility outcomes, providing insight into the clinical implications of the revised MUSA criteria.

## Materials and methods

### Study design and population

**Fig. 1:**
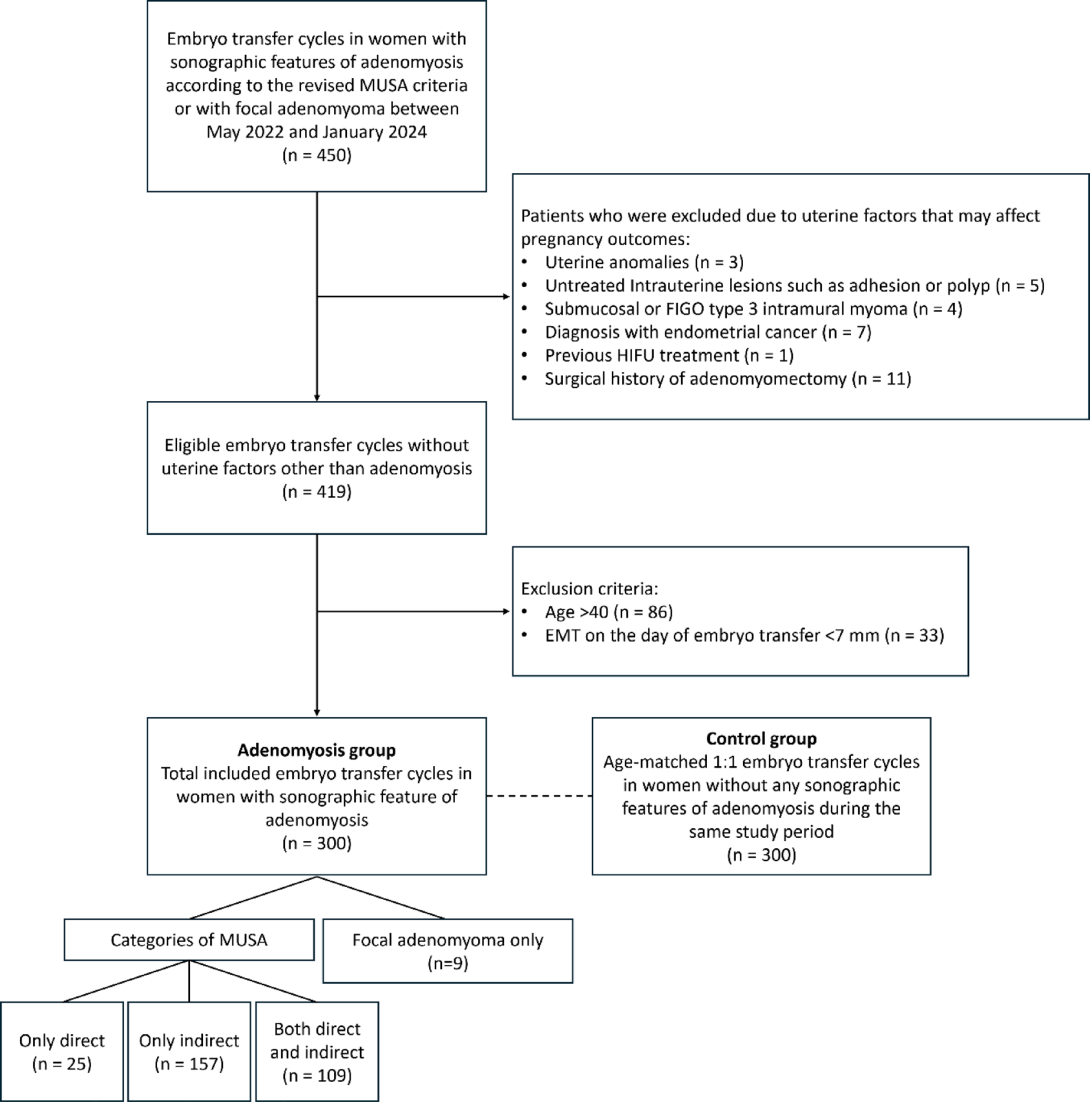
Flowchart summarizing inclusion and exclusion of embryo transfer cycles in the study.

This single-center retrospective cohort study analyzed data from IVF treatment cycles among women who underwent autologous embryo transfer between May 2022 and January 2024. A single expert in gynecologic ultrasound systematically examined the ultrasounds of all consecutive patients undergoing IVF according to the revised MUSA definition at the time of examination^7^. During the study period, women with at least one of the nine sonographic features of adenomyosis according to the revised MUSA criteria or with focal adenomyoma on ultrasound findings were assigned to the adenomyosis group (*n* = 450). Women with uterine factors other than adenomyosis that may affect pregnancy outcomes including uterine anomalies (*n* = 3), untreated intrauterine adhesion, endometrial polyp, submucosal myoma, or intramural myoma FIGO type 3 (*n* = 9), high-intensity focused ultrasound (HIFU) treatment (*n* = 1), previous diagnosis with endometrial cancer (*n* = 7), and surgical history of adenomyomectomy (*n* = 11) were xcluded from the analysis. Exclusion criteria included women older than 40 (*n* = 86), and endometrial thickness of less than 7 mm at the time of embryo transfer (*n* = 33). During the same period, a control group was selected from patients who underwent embryo transfer and had none of the sonographic features of adenomyosis. The control group was age-matched 1:1 with the adenomyosis group (Fig. 1).

To determine the appropriate sample size for our study, we aimed to detect a significant difference in clinical pregnancy rates (CPRs) between patients with and without specific ultrasound features of adenomyosis. Although no prior studies with an identical design were available for reference, a previous study reported pregnancy rate of 46.6% in women without adenomyosis feature per transfer, compared with 25.9% in women with at least one feature of adenomyosis^10^. Additionally, prior data showed that each of the MUSA features was observed in adenomyosis patients within a range of 15.1% to 57.7%^11^, and we assumed a prevalence of approximately 30%. Based on these data, we calculated the sample size required to detect a significant difference in CPR between patients with specific ultrasound features and those without. A total of 209 subjects were needed to achieve 80% power with a significance level of 0.05, using the difference in pregnancy rates to estimate the effect size. The sample size calculation was performed using G*power 3.1^12^.

### IVF/ET procedures

Ovarian stimulations and oocyte retrievals were performed under the standard protocol of our fertility center. Recombinant follicle-stimulating hormone (rFSH) and/or highly purified human menopausal gonadotropin (HP-hMG) was started on Day 3 of the menstrual cycle at an individualized dose based on the antral follicle count, anti-Müllerian Hormone (AMH), age, and predicted ovarian response^13^. For most patients, stimulation was commenced with a conventional gonadotropin dose (150–225 IU/day) in either agonist long or flexible antagonist protocol, whereas women at predicted risk of ovarian hyperstimulation syndrome (OHSS) received a reduced starting dose (100–150 IU/day). In selected cases, a higher dose of up to 300 IU/day was used at the discretion of the physician. Dose adjustments during mid-stimulation were generally avoided, as recommended by the European Society of Human Reproduction and Embryology (ESHRE) ovarian stimulation guideline^13^. Final oocyte maturation was triggered with 500 µg recombinant human chorionic gonadotropin (hCG) or 0.2 to 0.4 mg triptorelin when two or more follicles measuring 17–18 mm or larger were observed, and transvaginal ultrasound-guided oocyte retrieval was performed 34–36 h later. A freeze-all strategy was basically adopted in our center, so most cases involved frozen-thawed embryo transfer (FET); however, fresh ET was performed in 2.2% of the study cycles based on patient conditions or physician discretions. All cryopreserved embryos were frozen using vitrification. For the FETs, most cycles (88.1%) were conducted as programmed cycles. In the programmed cycles, 6 mg of oral estradiol valerate was administered daily from Day 3, and luteal phase support (LPS) was initiated when the endometrium reached a thickness of 7 mm. In the natural cycles, follicle size was monitored by ultrasound every 1–2 days starting on Day 10, and hCG triggering was performed when the dominant follicle reached 17–18 mm and the endometrium was thicker than 7 mm; LPS was started 2 days after triggering. For both fresh ET and FET, LPS included both subcutaneous and transvaginal progesterone administration and was maintained until 9–10 weeks of gestation if pregnancy was confirmed.

### Study outcomes

The primary outcome was CPR defined as the percentage of embryo transfers that resulted in an intra-uterine pregnancy confirmed by ultrasound. Other outcomes included the ongoing pregnancy rate (OPR), miscarriage rate (MR), and biochemical pregnancy loss rate (BPLR). Ongoing pregnancy rate was defined as the clinical pregnancy continuing beyond 12 weeks of gestation. Miscarriage was defined as a spontaneous loss of an intra-uterine pregnancy before 20 weeks of gestational age. BPLR was defined as the percentage of cycles of a positive serum beta hCG (≥ 5 mIU/mL) 9–10 days after the embryo transfer that resolved spontaneously without an intrauterine gestational sac on the ultrasound.

### Sonographic examination for evaluation of adenomyosis

**Fig. 2:**
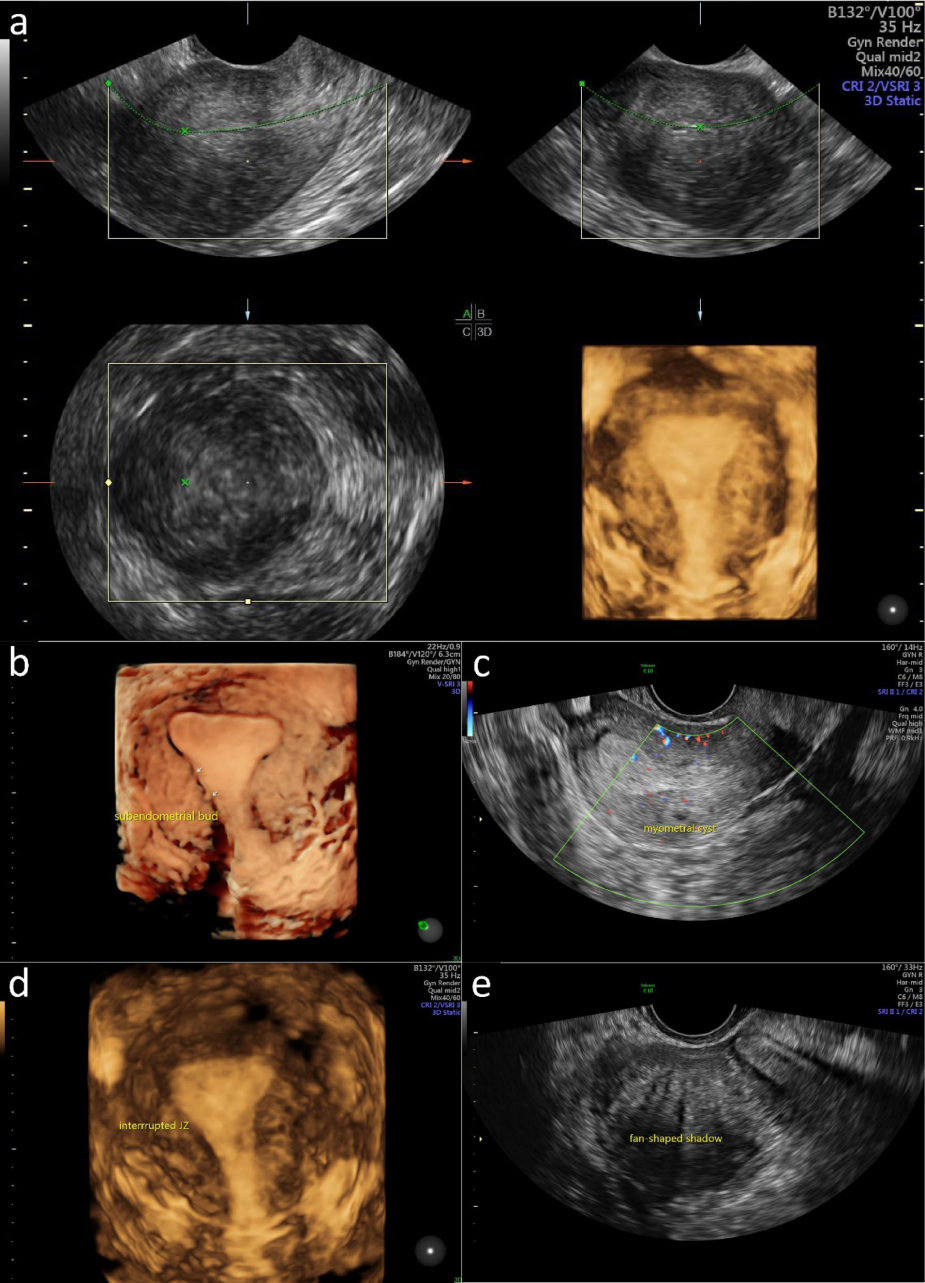
Transvaginal two-dimensional and three-dimensional ultrasound images of the patients in this study showing direct features (b, c) and indirect features (d, e) of adenomyosis as defined by the revised MUSA. (**a**) Acquisition of three-dimensional ultrasound images. (**b**) Subendometrial buds. (**c**) Myometrial cysts. (**d**) Interrupted junctional zone. (**e**) Fan-shaped shadowing.

All TVS ultrasound scans were performed in a standardized manner by the same imaging expert. (corresponding author, JY Lee, MD), utilizing the same Voluson 10 Expert BT19 (GE Healthcare, Wisconsin, USA) high-resolution ultrasound machine equipped with a 5–9 MHz transvaginal probe (RIC5-9D) for both 2D and 3D scans. 3D volumes were obtained with a single sweep wide angle (120°) in mid-quality settings, positioning the probe in the midsagittal plane. The rendered volume was adjusted to provide a coronal view of the uterus, capturing the endometrial-myometrial junction, myometrium, and interstitial tubes. Post-processing analysis of stored 2D and 3D images and cineloops was conducted at a later time. Various image optimization and postprocessing techniques are employed. Tomographic ultrasound imaging (TUI) for multislice imaging and volume contrast imaging (VCI) modes are utilized to evaluate the JZ across multiple planes^7^. In all patients, each of the three direct features and six indirect features of adenomyosis were assessed according to the revised MUSA definition (Fig. 2).

### Statistical analysis

All participants in the study had complete data for all variables and were thus fully included in the analysis. For the descriptive statistical analysis, continuous data are presented as the mean ± standard deviation (SD), and categorical data are presented as the number of cases with the corresponding percentages. The baseline characteristics between the groups were compared using t-tests for continuous variables and chi-square tests for categorical variables. Fisher’s exact test was used to compare pregnancy outcomes in patients with adenomyosis according to the category of MUSA features. Logistic regression analysis was performed to evaluate the odds ratios (ORs) of each variable including individual sonographic features of adenomyosis for pregnancy outcomes. The adjusted relative risks (aRRs) for clinical pregnancy, ongoing pregnancy, miscarriage, and biochemical pregnancy loss were determined and presented with 95% confidence intervals (CIs) using multivariable generalized estimating equation (GEE) models, which accounted for subjects contributing multiple cycles. Covariates were selected primarily based on prior clinical knowledge, as none of the baseline characteristics in the univariable analyses (Supplementary Table S1) reached statistical significance. Specifically, female age and AMH are well-recognized determinants of IVF success^14–16^, and the number of embryos transferred directly influences pregnancy outcomes^17^. PGT-A utilization was additionally included given its established impact on implantation rate and miscarriage rate per embryo transfer^18,19^. In all analyses, p-values less than 0.05 were considered statistically significant. All statistical analyses were performed using IBM SPSS Statistics for Windows, version 29.0 (IBM Corp., Armonk, NY, USA).

## Results

Among all autologous embryo transfer cycles performed during the study period, 300 embryo transfer cycles were included in the adenomyosis group. During the same period, an age-matched 1:1 control group was selected from women without sonographic features of adenomyosis.

The mean age was 36.1 ± 2.7 years in both groups, but the mean AMH level was significantly lower in the adenomyosis group than in the control group (2.8 ± 2.5 ng/mL vs. 3.3 ± 3.0 ng/mL, *p* = 0.015) (Table 1). Women in the adenomyosis group had a higher body mass index (BMI) than those in the control group, but the difference was not statistically significant (22.3 ± 3.5 vs. 21.9 ± 3.3, *p* = 0.095). Most cycles (97.8%) followed a freeze-all strategy with subsequent frozen-thawed embryo transfer, and 95.3% of cycles used the blastocysts-stage embryo, with an average of 1.5 ± 0.6 embryos transferred per cycle. Preimplantation genetic testing for aneuploidy (PGT-A) was utilized in 26.7% of all cycles with no significant difference in utilization rates between the two groups.

**Table 1:** Demographic and baseline characteristics of the study cycles.

Age (years)	Control (*N* = 300)	Adenomyosis (*N* = 300)	*p*-value
	36.1 ± 2.7	36.1 ± 2.7	0.750
BMI (kg/m^2^)	21.9 ± 3.3	22.3 ± 3.5	0.095
AMH (ng/ml)	3.3 ± 3.0	2.8 ± 2.5	0.015
Uterine volume (mL)	58.9 ± 21.2	85.3 ± 48.7	< 0.001
Nulliparous	242 (80.7)	235 (78.3)	0.479
Recurrent miscarriage	22 (7.3)	16 (5.3)	0.315
History of D&E	69 (23.0)	80 (26.7)	0.299
Once	52 (17.3)	59 (19.7)	0.462
More than twice	17 (5.7)	21 (7.0)	0.503
Primary IVF indication			0.012
Unexplained	143 (47.7)	169 (56.3)	
Ovulatory dysfunction	26 (8.7)	20 (6.7)	
Decreased ovarian reserve	55 (18.3)	57 (19.0)	
Tubal factor	9 (3.0)	17 (5.7)	
Male factor	44 (14.7)	27 (9.0)	
Combined	23 (7.7)	10 (3.3)	
Stage of embryo transferred			0.699
Cleavage	13 (4.3)	15 (5.0)	
Blastocyst	287 (95.7)	285 (95.0)	
PGT-A	86 (28.7)	74 (24.7)	0.268
Number of embryos transferred	1.5 ± 0.5	1.6 ± 0.6	< 0.001
EMT on the day of P start (mm)	9.2 ± 1.4	9.0 ± 1.6	0.366

Data are given as mean ± SD or n (%).

*AMH* anti-Müllerian hormone, *BMI* body mass index, *D&E* dilatation and evacuation, *EMT* endometrial thickness, *IVF* in vitro fertilization; *P* progesterone, *PGT-A* preimplantation genetic testing for aneuploidy.

For the ultrasound findings, the mean total number of MUSA features per woman was 2.5 ± 1.3, the mean number of direct features per woman was 0.5 ± 0.7, and the mean number of indirect features per woman was 1.9 ± 1.2. Fan-shaped shadowing was the most common MUSA feature, observed in 59.3% of cases, while subendometrial buds and lines were the least common, observed in 9.7% of cases (Supplementary Table S2).

The CPR and OPR were not different between the adenomyosis group and the age-matched control group overall (63.3% vs. 60.0%, *p* = 0.401; 49.7% vs. 50.7%, *p* = 0.806). The adenomyosis group had a higher MR than the control group, but the difference was not statistically significant (21.6% vs. 15.6%, *p* = 0.137). However, the BPLR was significantly higher in the adenomyosis group than in the control group (11.3% vs. 6.0%, *p* = 0.020). In the subgroup analysis based on the category of MUSA features, 157 patients had only indirect features, leading to an inconclusive diagnosis of adenomyosis, while 25 patients had only direct features and 109 patients had both direct and indirect features. There were no differences in baseline characteristics (Supplementary Table S3), and no significant differences in CPR, OPR, MR, and BPLR between the subgroups (Table 2).

**Table 2:** Pregnancy outcomes in the control group and adenomyosis group according to the category of MUSA features.

Clinical pregnancy (%)	Control (*n* = 300)	Adenomyosis (*n* = 300)	Only direct features (*n* = 25)	Only Indirect features (*n* = 157)	Both direct and indirect features (*n* = 109)
	60.0 (180/300)	63.3 (190/300)	68.0 (17/25)	59.9 (94/157)	65.1 (71/109)
Ongoing pregnancy (%)	50.7 (152/300)	49.7 (149/300)	64.0 (16/25)	43.9 (69/157)	53.2 (58/109)
Biochemical pregnancy loss (%)	6.0 (18/300)^*^	11.3 (34/300)^*^	4.0 (1/25)	14.6 (23/157)	8.3 (9/109)
Miscarriage (%)	15.6 (28/180)	21.6 (41/190)	5.9 (1/17)	26.6 (25/94)	18.3 (13/71)

*P < 0.05.

**Fig. 3:**
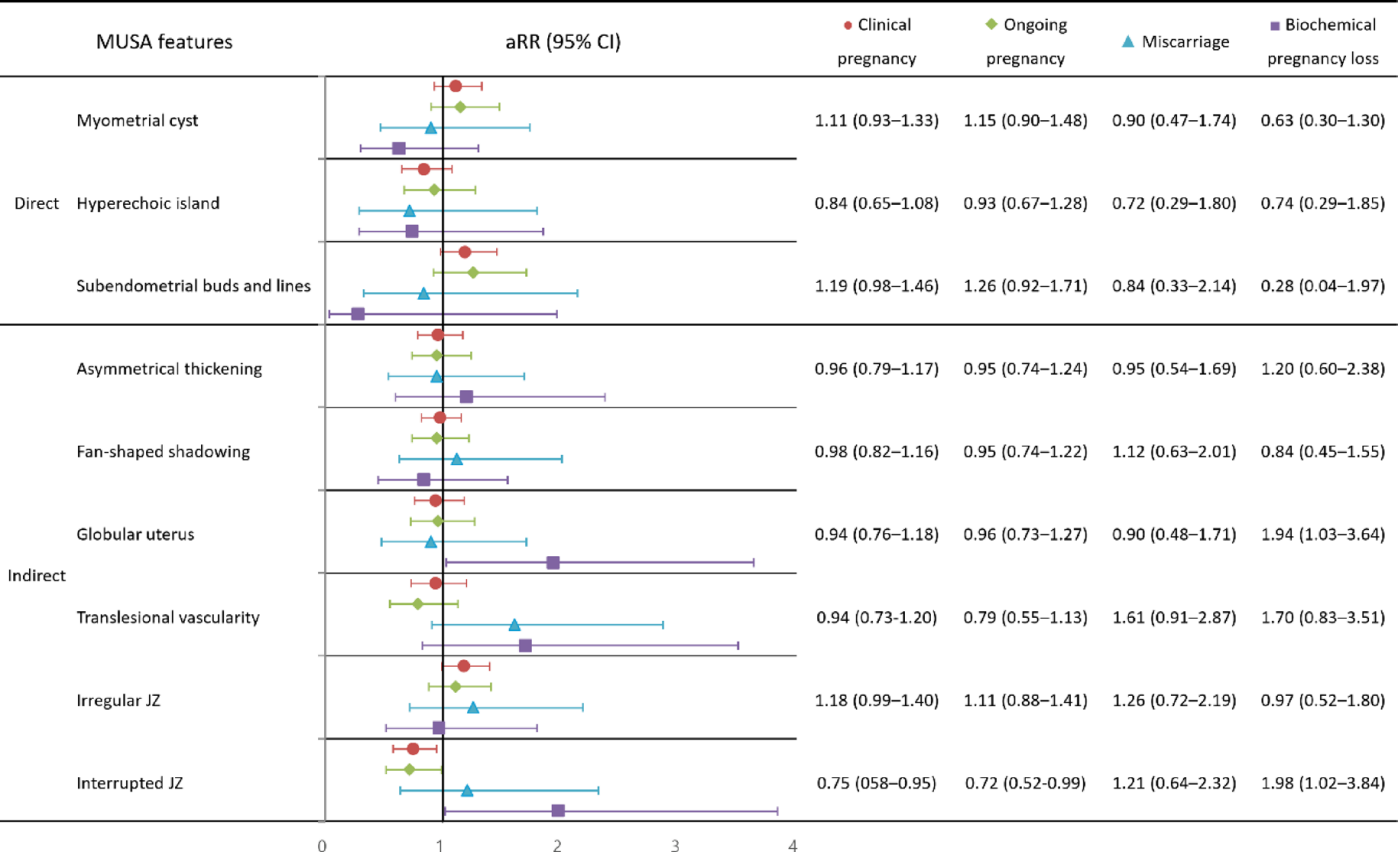
Adjusted relative risks (aRRs) of individual MUSA features for clinical pregnancy, ongoing pregnancy, biochemical pregnancy loss, and miscarriage after adjusting for age, AMH, number of embryos transferred, and PGT-A utilization.

Among the MUSA features, interrupted JZ was the only independent factor that was significantly associated with CPR and OPR with the odds ratio (OR) of 0.50 (95% CI 0.28–0.88) and 0.52 (95% CI 0.30–0.93), respectively (Supplementary Table S1). The utilization of PGT-A was significantly associated with a higher chance of ongoing pregnancy and a lower risk of miscarriage (Supplementary Table S1). After adjusting for age, AMH, number of embryos transferred, and PGT-A utilization, patients with the interrupted JZ had a significantly lower risk of achieving clinical pregnancy (adjusted RR (aRR) = 0.75, 95% CI 0.58–0.95, *p* = 0.020), ongoing pregnancy (aRR = 0.72, 95% CI 0.52–0.99, *p* = 0.049), and an increased risk of biochemical pregnancy loss (aRR = 1.98, 95% CI 1.02–3.84, *p* = 0.044). However, there was no significant increase in the risk of miscarriage after clinical pregnancy (aRR = 1.21, 95% CI 0.64–2.32, *p* = 0.557) (Fig. 3).

## Discussion

This study aimed to evaluate the clinical relevance of individual ultrasound features in the revised MUSA criteria for adenomyosis. Our findings indicate that patients with an interrupted JZ had approximately a 30% lower probability of achieving clinical pregnancy and ongoing pregnancy, as well as a twofold increased risk of biochemical pregnancy loss. However, once a gestational sac is observed, the miscarriage risk is not significantly influenced by JZ characteristics, suggesting that an interrupted JZ primarily impacts early pregnancy stages, including decidualization and implantation.

The interrupted JZ is classified as an indirect feature in the MUSA consensus, indicating that adenomyosis cannot be diagnosed if only indirect features are present. Our study identified 157 cases exhibiting only indirect features (Table 2). Importantly, no significant differences in fertility outcomes were found between cases with direct features alone and those with both direct and indirect features. This raises concerns that cases with only indirect features but compromised fertility may go undetected under the current revised diagnostic criteria.

The 2022 MUSA guidelines recommend evaluating the JZ using 3D ultrasound in coronal, sagittal, and transverse views, classifying it as an indirect feature^7^. However, from a pathophysiological perspective, the JZ plays a key role in endometrial infiltration into the myometrium, aligning with direct criteria in clinical significance. Bergeron et al. proposed that adenomyosis involves basal endometrium invasion through an altered or absent JZ^20^. In addition, molecular studies have demonstrated dysregulation of implantation-associated factors, including HOXA10, LIF, MMP2, IL-6, cytochrome P450, and RCAS1, leading to reduced endometrial receptivity and impaired decidualization in women with adenomyosis^21–23^.

Several studies have linked increased JZ thickness to poor fertility outcomes, including implantation failure in IVF, with rates as high as 95.8% for JZ thickness > 7 mm^24^. These findings, primarily MRI-based, associate thicker JZ with miscarriage, using diagnostic thresholds between 7 and 12 mm^24–27^. Another study found a strong correlation between infertility and five or more adenomyotic findings but did not assess individual sonographic features or follow MUSA criteria^28^. Similarly, Cozzolino et al. linked multiple JZ abnormalities to miscarriage risk; however, their study was conducted prior to the publication of the revised MUSA criteria and did not analyze individual features^29^.

Dason et al. reported that under the revised MUSA criteria, adenomyosis was diagnosed in 76% of cases compared to 22% using previous criteria, with all newly diagnosed cases exhibiting an interrupted or irregular JZ^6^. In our study, 38.3% and 20.7% of diagnosed cases exhibited interrupted or irregular JZ, respectively. The reasons for this discrepancy are not entirely clear and are likely multifactorial, potentially reflecting differences in study population characteristics, referral patterns, and the criteria applied. Notably, the adenomyosis prevalence reported by Dason et al. (76%) is considerably higher than the approximately 30% prevalence reported in prior studies^30^, which may also be influenced by population-specific factors or differences in scanning protocols. Unlike previous studies linking JZ abnormalities to miscarriage^26,27^, our findings indicate that JZ irregularities mainly affect implantation rather than later pregnancy stages. This distinction is crucial, as most previous studies were MRI-based, whereas our study adhered strictly to the revised MUSA definitions without including JZ thickness. The revised guidelines removed JZ thickness measurement due to insufficient clinical evidence. However, given our findings, reconsideration of its exclusion as an ultrasound feature may be warranted.

Supporting our findings, a prior study on IVF outcomes identified interrupted JZ as the only ultrasound feature significantly associated with lower cumulative live birth rates (CLBRs)^10^. However, our study differs in several key aspects: we analyzed each embryo transfer cycle as an independent case, allowing for precise evaluation of adenomyosis’ impact on implantation and endometrial receptivity. Additionally, we adjusted for critical confounders, including embryo stage and number, which were not accounted for in previous studies.

Interestingly, our study also found that women with adenomyosis had significantly lower AMH levels than age-matched controls, consistent with Yin et al.‘s findings of impaired ovarian reserve, reduced AFC, and diminished ovarian blood perfusion in adenomyosis patients^31^. Excessive estrogen and progesterone resistance may contribute to ovarian impairment by suppressing androgen levels and reducing vascular endothelial growth factor and insulin-like growth factor expression, leading to decreased ovarian perfusion^32^. Although the observed association between decreased AMH and adenomyosis is biologically plausible, a causal relationship cannot be established due to the retrospective observational design of this study.

Focal adenomyosis, not included in MUSA, was analyzed separately and showed no significant association with clinical pregnancy (Supplementary Table S1). Further research is needed to determine whether focal adenomyosis influences fertility when combined with other direct and indirect features.

This study has several strengths. First, all ultrasound examinations were conducted by a single expert following the MUSA criteria, ensuring diagnostic consistency. Second, our sample size is the largest among studies assessing individual sonographic features based on the revised MUSA criteria. Third, by analyzing individual embryo transfer cycles and adjusting for embryonic factors, we provide a robust evaluation of the impact of adenomyosis on implantation and endometrial receptivity. However, there are several limitations to consider. As this study was conducted on patients visiting fertility centers, applying the results to the general population of reproductive-aged women may be limited. Additionally, although confounding factors were carefully considered and adjusted for using GEE, potential bias should be acknowledged. Another limitation is the challenge of JZ assessment via ultrasound, as technical constraints may affect diagnostic accuracy, as mentioned in the revised MUSA criteria^7^. However, all ultrasounds were performed by a single expert to ensure consistency. Additionally, adenomyosis severity and extrinsic effects were not classified, as these are not included in the revised MUSA guidelines, highlighting the need for further research. Lastly, due to the nature of fertility centers, most patients were transferred to obstetric hospitals for delivery, making it challenging to follow up with all patients through to delivery. Future studies with long-term follow-up could provide further insight into the clinical implications of adenomyosis on fertility outcomes.

Our findings suggest that the presence of an interrupted junctional zone is significantly associated with reduced clinical and ongoing pregnancy rates and an increased risk of biochemical pregnancy loss. These results emphasize the importance of JZ assessment in diagnosing adenomyosis, particularly in predicting embryo implantation success. Given its clinical significance, JZ disruption should be given greater weight in adenomyosis diagnosis, especially in reproductive-aged women. Future research should further refine the role of JZ abnormalities in fertility and explore their implications for clinical practice.

## Supplementary Information

Below is the link to the electronic supplementary material.

Supplementary Material 1

## Data Availability

The data underlying this article will be shared on reasonable request to the corresponding author.
