# Supplementary material for: Association of revised morphological uterus sonographic assessment (MUSA) features of adenomyosis and IVF outcomes
Source: Sci Rep. 2026 Apr 30;16:20034. doi: 10.1038/s41598-026-50999-w (PMC13319446; doi:10.1038/s41598-026-50999-w)
Supplement: Supplementary file 1 — Supplementary Material 1 [file 41598_2026_50999_MOESM1_ESM.docx]

**Supplementary Table S1.** Univariable logistic regression for pregnancy outcomes

|  | Clinical pregnancy | | Ongoing pregnancy | | Miscarriage | | Biochemical pregnancy loss | |
| --- | --- | --- | --- | --- | --- | --- | --- | --- |
|  | Crude OR | 95% CI | Crude OR | 95% CI | Crude OR | 95% CI | Crude OR | 95% CI |
| Age | 0.99 | 0.90–1.08 | 0.95 | 0.87–1.03 | 1.11 | 0.97–1.28 | 1.02 | 0.90–1.17 |
| BMI | 0.99 | 0.93–1.06 | 0.95 | 0.89–1.01 | 1.10 | 0.99–1.20 | 1.05 | 0.96–1.16 |
| AMH | 1.09 | 0.98–1.20 | 1.05 | 0.96–1.15 | 1.01 | 0.89–1.14 | 1.04 | 0.91–1.18 |
| Uterine volume (mL) | 1.00 | 0.99–1.01 | 1.00 | 0.99–1.00 | 1.00 | 0.99–1.01 | 0.99 | 0.99–1.01 |
| Recurrent miscarriage | 0.56 | 0.20–1.54 | 0.59 | 0.21–1.67 | 1.22 | 0.24–6.29 | 1.88 | 0.51–6.98 |
| PGT-A | 1.28 | 0.73–2.23 | 1.82 | 1.07–3.11 | 0.32 | 0.12–0.87 | 1.11 | 0.49–2.51 |
| EMT on the day of P start | 0.94 | 0.81–1.09 | 0.98 | 0.85–1.13 | 0.95 | 0.75–1.20 | 1.15 | 0.94–1.41 |
| Number of embryos transferred | 1.21 | 0.79–1.83 | 0.96 | 0.65–1.44 | 1.61 | 0.82–3.15 | 0.75 | 0.39–1.43 |
| Direct features | 1.20 | 0.75–1.93 | 1.50 | 0.95–2.37 | 0.53 | 0.26–1.08 | 0.48 | 0.22–1.04 |
| Indirect features | 0.59 | 0.26–1.31 | 0.50 | 0.24–1.05 | 2.19 | 0.62–7.73 | 2.19 | 0.50–9.57 |
| Myometrial cyst | 1.33 | 0.80–2.20 | 1.33 | 0.80–2.20 | 0.72 | 0.33–1.60 | 0.64 | 0.27–1.52 |
| Hyperechoic island | 0.87 | 0.47–1.63 | 0.87 | 0.47–1.63 | 0.62 | 0.20–1.93 | 0.69 | 0.23–2.06 |
| Subendometrial buds and lines | 1.67 | 0.72–3.89 | 1.59 | 0.74–3.43 | 0.79 | 0.25–2.47 | 0.25 | 0.03–1.88 |
| Asymmetrical thickening | 0.88 | 0.52–1.48 | 0.86 | 0.52–1.43 | 1.13 | 0.53–2.42 | 1.24 | 0.58–2.67 |
| Fan-shaped shadowing | 0.90 | 0.56–1.46 | 0.83 | 0.52–1.31 | 1.31 | 0.64–2.66 | 0.85 | 0.42–1.75 |
| Globular uterus | 0.85 | 0.50–1.46 | 0.88 | 0.53–1.49 | 1.01 | 0.45–2.27 | 2.00 | 0.95–4.21 |
| Translesional vascularity | 0.94 | 0.52–1.69 | 0.79 | 0.44–1.40 | 1.53 | 0.67–3.50 | 1.59 | 0.70–3.63 |
| Irregular JZ | 1.53 | 0.95–2.55 | 1.25 | 0.78–1.99 | 1.25 | 0.62–2.50 | 1.00 | 0.48–2.08 |
| Interrupted JZ | 0.50 | 0.28–0.88 | 0.52 | 0.30–0.93 | 1.33 | 0.55–3.24 | 2.02 | 0.92–4.40 |
| Focal adenomyoma | 1.63 | 0.89–2.99 | 1.65 | 0.94–2.88 | 0.71 | 0.30–1.67 | 1.15 | 0.50–2.69 |
| Number of features | 0.96 | 0.80–1.15 | 0.94 | 0.79–1.12 | 1.06 | 0.83–1.36 | 1.07 | 0.82–1.40 |

AMH, anti-Müllerian hormone; BMI, body mass index; D&E, dilatation and evacuation; EMT, endometrial thickness; JZ, junctional zone; P, progesterone; PGT-A, preimplantation genetic testing for aneuploidy.

**Supplementary Table S2.** Distribution of MUSA features in patients with adenomyosis

|  | Adenomyosis  (N = 300) |
| --- | --- |
| Direct features |  |
| Myometrial cyst | 84 (28.0) |
| Hyperechoic island | 47 (15.7) |
| Subendometrial buds and lines | 29 (9.7) |
| Indirect features |  |
| Asymmetrical thickening | 85 (28.3) |
| Fan-shaped shadowing | 178 (59.3) |
| Globular uterus | 76 (25.3) |
| Translesional vascularity | 58 (19.3) |
| Irregular junctional zone | 115 (38.3) |
| Interrupted junctional zone | 62 (20.7) |
| Direct features only | 25 (8.3) |
| Focal adenomyoma | 63 (21.0) |

Data are given as n (%).

**Supplementary Table S3.** Baseline characteristics of subgroups according to the category of MUSA features

|  | Only direct features  (n = 25) | Only indirect features  (n = 157) | Both direct and indirect features  (n = 109) |
| --- | --- | --- | --- |
| Age (years) | 35 (34–37) | 37 (35–38.5) | 36 (35–38) |
| BMI (kg/m^2^) | 21.9 (19.8–23.5) | 21.4 (20.4–24.4) | 21.5 (19.9–23.9) |
| AMH (ng/ml) | 2.8 (1.1–3.6) | 2.1 (1.3–3.8) | 1.7 (1.0–2.9) |
| History of D&E | 5 (20.0) | 42 (26.8) | 32 (29.4) |
| PGT-A | 4 (16.0) | 36 (22.9) | 32 (29.4) |
| EMT on the day of P start (mm) | 8.7 (8.1–10.0) | 8.6 (7.9–9.6) | 8.5 (8.1–10.1) |
| Number of embryos transferred | 2 (1–2) | 2 (1–2) | 2 (1–2) |

Data are given as median (interquartile range), or n (%). AMH, anti-Müllerian hormone; BMI, body mass index; D&E, dilatation and evacuation; EMT, endometrial thickness; P, progesterone; PGT-A, preimplantation genetic testing for aneuploidy.
